# 双免疫联合治疗在晚期非小细胞肺癌中的一线应用

**DOI:** 10.3779/j.issn.1009-3419.2021.102.48

**Published:** 2022-02-20

**Authors:** 海怡 邓, 李强 王, 伊霖 杨, 建辉 吴, 承志 周

**Affiliations:** 510120 广州，呼吸疾病国家重点实验室，国家呼吸系统疾病临床研究中心，广州医科大学附属第一医院，广州呼吸健康研究院 State Key Laboratory of Respiratory Disease, National Clinical Research Center for Respiratory Disease; Guangzhou Institute of Respiratory Health, The First Affiliated Hospital of Guangzhou Medical University, Guangzhou 510120, China

**Keywords:** 肺肿瘤, 双免疫联合治疗, PD-1抑制剂, CTLA-4抑制剂, 生物标志物, Lung neoplasms, Dual immunotherapy, PD-1 inhibitor, CTLA-4 inhibitor, Biomarker

## Abstract

近年来，程序性死亡受体1（programmed death protein-1, PD-1）/程序性死亡配体1（programmed death-ligand 1, PD-L1）抑制剂单药、PD-1抑制剂联合化疗日益广泛地用于非小细胞肺癌（non-small cell lung cancer, NSCLC）的一线治疗，但PD-L1低表达的患者治疗反应和生存获益有限，现有治疗方案尚不能完全满足真实世界患者的临床需求。因此，研究者们仍在探索新的有效或者高效的治疗方案，以求进一步提高NSCLC不同人群的疗效和生存预后。双免疫联合治疗，如PD-1抑制剂和细胞毒性T淋巴细胞相关抗原4（cytotoxic T lymphocyte associated antigen-4, CTLA-4）抑制剂联合在多种肿瘤中均显示出可观的长期生存获益，在NSCLC中亦显示出广阔的应用前景。除探索不同的新兴联合方案外，如何通过生物标志物准确识别最佳获益人群、如何通过多学科协作有效管理联合方案的安全性，也是双免疫联合治疗受关注的重点。本文就双免疫联合治疗的作用机制、研究进展、疗效预测标志物及未来探索方向等作一综述。

## Abstract (en)

Programmed cell death protein-1 (PD-1)/programmed cell death-ligand 1 (PD-L1) inhibitors and PD-1 inhibitors plus chemotherapy combination regimens have been widely used in the first-line treatment of advanced non-small cell lung cancer(NSCLC), but patients with low PD-L1 expression have limited objective response and survival benefits. Existing treatment regimens are still difficult to fully meet the clinical needs of patients in the real world. Therefore, researchers are still exploring novel superactive treatment options to further improve the efficacy and survival prognosis of different sub-groups in NSCLC. Dual immunotherapy [such as the combination of PD-1 and cytotoxic T lymphocyte associated antigen-4 (CTLA-4) inhibitors] has shown considerable long-term survival benefits in a variety of tumors and has also shown broad clinical prospects in NSCLC. In addition to exploring different emerging combination options, how to accurately identify the optimal-benefit groups through predictive biomarkers and how to effectively manage the safety of combination immunotherapy through multidisciplinary collaboration are also the focus of dual immunotherapy. This article reviews the mechanism of action, research progress, predictive biomarkers and future exploration directions of dual immunotherapy.

近年来免疫治疗的研究进展使非小细胞肺癌（non-small cell lung cancer, NSCLC）的治疗格局日新月异。国家药品监督管理局批准程序性死亡受体/配体1（programmed cell death protein/ligand 1, PD-1/PD-L1）抑制剂单药、PD-1抑制剂联合化疗用于NSCLC的一线治疗。临床研究^[1]^表示PD-L1高表达的患者更容易从免疫治疗中获益；但对于PD-L1低表达或者阴性的患者，免疫治疗带来的生存获益改善有限，现有的治疗方案并不能满足真实世界这类患者的需求。探索新的有效或者高效的治疗方案对进一步提高NSCLC不同人群的疗效和预后至关重要。双免疫联合治疗[如PD-1抑制剂和细胞毒性T淋巴细胞相关抗原4（cytotoxic T lymphocyte associated antigen-4, CTLA-4）抑制剂]在黑色素瘤、肾细胞癌、结直肠癌、肝细胞癌中已经展现了可观的长期生存获益^[1]^。近期临床研究^[2, 3]^证实双免疫联合治疗在NSCLC中有广阔的应用前景。本文就双免疫联合治疗的作用机制和研究进展以及疗效预测标志物作一综述。

## 1 双免疫联合治疗的作用机制

PD-1与CTLA-4同属免疫检查点分子，但作用机制不同，在免疫反应的不同阶段对T细胞的活化发挥负向调控作用^[4]^。CTLA-4可在初始T细胞的最初活化阶段阻止T细胞的激活与效应功能，而PD-1则在免疫反应的较晚期作用于活化T细胞，抑制T细胞的活化程度与细胞毒性^[4]^。研究提示，PD-1抑制剂与CTLA-4抑制剂的联合并非简单的相加，其叠加效应远远大于两者单独应用时的效应总和。两者联合应用时，可显著减少耗竭表型的细胞毒性CD8^+^ T细胞比例，增加活性效应T细胞的存在比例（包括活性CD8^+^和CD4^+^效应细胞）^[5]^。有关耗竭T细胞的“消失”机制尚未明确，一种推测是联合治疗直接将耗竭T细胞转化为活性效应细胞；另一种推测为耗竭T细胞凋亡后被新生的活化CD8^+^效应细胞所替代。尽管耗竭T细胞仍可在“疲劳”状态下工作，但工作效率即杀伤力将显著降低，因此，免疫细胞群从耗竭T细胞向活性效应细胞的构成转变可在很大程度上增强总体免疫反应^[5]^。

临床前研究亦证实PD-1抑制剂和CTLA-4抑制剂联合治疗可在小鼠模型中显著降低肿瘤的复发和转移风险，从而显著延长小鼠生存期（*P* < 0.05）^[6]^。而其潜在机制可能为协同刺激CD8^+^ T细胞和CD4^+^ T细胞浸润至肿瘤微环境（与各抑制剂单独用药相比，T细胞浸润比例增加*P* < 0.05），从而刺激抗肿瘤免疫效应^[6]^。

## 2 双免疫治疗在NSCLC中的应用

双免疫治疗方案，主要是PD-1抑制剂纳武利尤单抗（Nivolumab, N）+CTLA-4抑制剂伊匹木单抗（Ipilimumab, I）联合方案在晚期NSCLC患者中取得生存获益。

### 2.1 早期剂量探索研究

关于Nivolumab与Ipilimumab的联合用药探索起始于黑色素瘤中的研究（包括CA209-004、CheckMate 067、069等），Ⅰ期剂量探索研究CA209-004（*n*=86）显示Nivolumab+Ipilimumab同时给药方案疗效显著优于Ipilimumab、Nivolumab序贯给药。在不同剂量组合的同时用药联合方案中，1 mg/kg Nivolumab+3 mg/kg Ipilimumab（N1+I3）、3 mg/kg Nivolumab+1 mg/kg Ipilimumab（N3+I1）和3 mg/kg Nivolumab+3 mg/kg Ipilimumab（N3+I3）组患者的客观缓解率（objective response rate, ORR）和总临床有效率达到预期，ORR分别为53%、40%和50%，总临床有效率分别为65%、73%和83%。但N1+I3及N3+I3方案因3级以上治疗相关不良反应发生率高，分别达到和超过了最大耐受剂量（N1+I3组：3级葡萄膜炎、3级谷丙转氨酶/谷草转氨酶水平升高各1例；N3+I3组：3/6例患者出现3级-4级脂肪酶水平升高且持续时间≥3周）^[7]^。此后的临床研究又陆续探索了Nivolumab与小剂量Ipilimumab在其他瘤种的联合方案。Ipilimumab是一种IgG1亚型的全人源单克隆抗体，该亚型单抗具有较强的抗体依赖性细胞介导的细胞毒（antibody-dependent cell-mediated cytotoxicity, ADCC）活性^[8]^。大多数抗PD-1/PD-L1制剂无ADCC作用，在机体中表现为纯拮抗作用，超过一定剂量（如3 mg/kg常规剂量）后不显著提高疗效亦不会显著增加导致治疗中止或死亡的安全性事件，治疗窗较广^[9]^，而Ipilimumab的疗效和安全性与剂量均呈明显相关性^[10]^。

CheckMate 012研究首次探索了N+I双免疫联合治疗不同剂量组合方案在晚期NSCLC患者中的有效性和安全性，基于该研究结果确定了NSCLC患者中Nivolumab+Ipilimumab的最佳联合方案与剂量。该研究双免疫联合探索的附加队列中包含了三种剂量组合：1 mg/kg Nivolumab *Q2W*联合1 mg/kg Ipilimumab *Q6W*（N1+I1）、3 mg/kg Nivolumab *Q2W*联合1 mg/kg Ipilimumab *Q12W*（N3+I1, *Q12W*）与3 mg/kg Nivolumab *Q2W*联合1 mg/kg Ipilimumab *Q6W*（N3+I1, *Q6W*），治疗直至疾病进展、发生不可耐受的毒性反应或撤回知情同意。结果提示N3+I1 *Q6W*联合方案具有良好的耐受性且不影响疗效^[11]^；且肿瘤生长动态模型预测该方案可较其他任一方案更强效地遏制肿瘤生长并使肿瘤体积缩小，因此N3+I1 *Q6W*被选作胸部肿瘤后续Ⅱ期临床研究的剂量方案^[12]^。

### 2.2 Ⅱ期研究

Ⅱ期研究CheckMate 568第一部分纳入了288例初治的晚期NSCLC患者，并根据PD-L1表达及肿瘤突变负荷（tumor mutation burden, TMB）对患者进行了分层分析，治疗方案为3 mg/kg Nivolumab *Q2W*联合1 mg/kg Ipilimumab *Q6W*。研究主要终点是PD-L1≥1%或 < 1%患者的ORR，次要终点为基于TMB分层的有效性。结果显示，双免疫治疗（N3+I1）在总体人群中的ORR达30%，且治疗反应持久存在^[13]^。PD-L1≥1%的患者亚组ORR及中位无进展生存期（progression-free survival, PFS）均显著优于PD-L1 < 1%的患者（ORR：41% *vs* 15%；中位PFS：6.8个月*vs* 2.8个月）；高TMB的患者（TMB≥10突变/Mb）也显著优于低TMB的患者（ORR：44% *vs* 12%；中位PFS：7.1个月*vs* 2.6个月）。双免疫方案的不良反应谱与既往临床研究类似，≥3级的治疗相关不良事件（treatment related adverse event, TRAE）发生率为29%，16%的患者因TRAE中止治疗^[13]^。值得注意的是，与其他黑色素瘤或NSCLC中CTLA-4和/或PD-1抑制剂的结果类似，双免疫治疗方案与化疗组的生存曲线出现交叉或延迟分离，即研究初期化疗组生存预后优于或类似于双免疫联合治疗组，但双免疫联合治疗组的长期生存预后显著优于化疗，且优势随随访时间延长而扩大。因此，按照常规等比例风险模型进行统计学分析时[如*Kaplan-Meier*法计算的中位总生存期（overall survival, OS）]，双免疫联合治疗的延迟临床效应可能在统计学效力上有所削弱^[14]^。

CheckMate 568研究^[15]^第二部分则评估了双免疫治疗联合化疗的可行性，并完成了适宜联合方案的剂量探索。研究共纳入36例初治晚期NSCLC患者，97%的患者完成了2个周期含铂二联化疗+双免疫联合治疗（N, 360 mg, *Q3W*+I1, *Q6W*），最短随访26个月时，≥3级TRAE发生率为58%（21例），22%（8例）的患者因TRAE导致停药，主要的停药原因为结肠炎、脑病、肺炎和关节痛。双免疫治疗联合化疗的中位OS和中位PFS分别为19.4个月和10.8个月，1年和2年的OS率分别为61%和43%，1年和2年的PFS率分别为42%和24%，ORR为47%，89%的患者达到疾病控制^[15]^。

### 2.3 Ⅲ期研究

#### 2.3.1 双免疫联合治疗（N+I）

CheckMate 227是一项开放、随机Ⅲ期临床研究^[16]^。研究根据PD-L1表达（≥1%或 < 1%）分层，分层后人群各随机分为三组：①PD-L1≥1%人群（*n*=1, 189）：Nivolumab（3 mg/kg, *Q2W*）+Ipilimumab（1 mg/kg, *Q6W*）组、化疗组、Nivolumab（240 mg, *Q2W*）组；②PD-L1 < 1%人群（*n*=550）：Nivolumab+Ipilimumab、化疗组、Nivolumab（360 mg, *Q3W*）+化疗组。各对照组的入组患者基线特征均平衡，其中鳞癌患者占28%，非鳞癌患者占72%。68%的患者PD-L1表达水平≥1%。在可评估肿瘤负荷的患者中，高TMB的患者占44%（双免疫联合组*vs*化疗组：42% *vs* 46%）。2年随访结果显示，PD-L1≥1%的患者双免疫治疗组和化疗组的中位OS分别为17.1个月和14.9个月，联合治疗组死亡风险显著降低21%[风险比（hazard ratio, HR）=0.79，*P*=0.007]^[16]^。PD-L1≥1%人群，双免疫治疗组和化疗组的3年OS率分别为33%和22%。值得注意的是，在双免疫治疗组确认有治疗反应的患者中，38%在3年后仍有治疗反应，而这一患者比例在化疗组中仅为4%^[17]^。联合治疗组与化疗组患者的中位持续缓解时间分别为23.2个月和6.7个月。因此，对于有治疗反应的患者，双免疫联合治疗的缓解可持续性显著增强。

PD-L1 < 1%人群的数据同样值得关注。最短随访时间为29.3个月时，PD-L1 < 1%的患者双免疫治疗组对比化疗组中位OS分别为17.2个月和12.2个月（HR=0.62）^[16]^，3年OS率分别为34%和15%^[17]^。双免疫治疗组中获得疾病缓解的患者中，34%在3年后仍持续缓解，而化疗组为0%^[17]^。这一数据对于PD-L1阴性人群意义重大，值得更大样本量人群的证实，并深入探索其中的潜在机制。

CheckMate 227最新公布的4年随访数据再次在NSCLC中验证了N+I双免疫治疗为晚期肿瘤患者带来的持续缓解和长期生存获益^[18]^。双免疫治疗组和化疗组总体人群的4年OS率分别为27%和15%（其中PD-L1≥1%人群：29% *vs* 18%；PD-L1 < 1%人群：24% *vs* 10%）、4年PFS率分别为14%和3%、4年缓解持续（duration of response, DOR）率分别为33%和6%^[18]^。就组织学分型而言，鳞癌与非鳞癌患者均可从双免疫治疗中获益，但鳞癌患者生存获益似乎更为显著：PD-L1≥1%人群中，鳞癌与非鳞癌患者的OS HR分别为0.68（双免疫联合组*vs*化疗组：14.8个月*vs* 9.2个月）和0.81（19.4个月*vs* 17.2个月）；PD-L1 < 1%人群中，鳞癌与非鳞癌患者OS的HR分别为0.53（双免疫联合组*vs*化疗组：15.9个月*vs* 8.5个月）和0.69（17.5个月*vs* 13.1个月）^[18]^。总的来说，无论患者PD-L1表达或组织学分型如何，双免疫治疗组患者的4年OS、PFS和DOR相关的获益均持续存在^[18]^。

基于NSCLC中双免疫临床研究结果，2020年5月，美国食品药品监督管理局批准Nivolumab联合Ipilimumab一线用于PD-L1≥1%的转移性NSCLC患者^[2]^。

#### 2.3.2 双免疫联合治疗（固定剂量N+I）

CheckMate 817是一项多中心、开放性Ⅲb期/Ⅳ期研究，分别在一般人群[体能状态（performance status, PS）评分≤1分，*n*=391]和特殊人群（PS=2分或PS=0分-1分但有合并症的患者，*n*=198）中探索了Nivolumab（240 mg，*Q2W*固定剂量）联合Ipilimumab（1 mg/kg, *Q6W*）的安全性和疗效^[19, 20]^。结果显示，一般人群和特殊人群固定剂量N+I方案的ORR分别为36%和24%（其中PS=2分人群ORR为19%；PS评分0分-1分伴合并症人群37%），中位PFS分别为5.8个月和3.9个月^[20]^。两人群中高TMB和高PD-L1表达的患者生存预后均较好（一般人群中TMB≥10 *vs* TMB < 10的PFS：10.9个月*vs* 4.2个月；PD-L1≥50% *vs* PD-L1 < 1%的PFS：8.4个月*vs* 5.3个月；特殊人群中TMB≥10 *vs* TMB < 10的PFS：8.3个月*vs* 2.8个月；PD-L1≥50% *vs* PD-L1 < 1%的PFS：9.6个月*vs* 3.9个月）^[20]^。固定剂量N+I方案安全性谱与按体重调节剂量方案一致，毒性可控。一般人群任意级别和3级-4级TRAE的发生率分别为77%和35%，特殊人群为67%和28%。两人群分别有22%和15%的患者因TRAE中止治疗^[20]^。

#### 2.3.3 双免疫联合治疗（N+I）+化疗

CheckMate 9LA研究是一项开放性、多中心、随机Ⅲ期临床研究（*n*=719）^[21]^，比较单用化疗（最多4个周期，随后进行可选的培美曲塞维持治疗；*n*=358）和Nivolumab（360 mg, *Q3W*）+Ipilimumab（1 mg/kg, *Q6W*）联合化疗（2个周期；*n*=361）一线治疗晚期NSCLC患者（无论PD-L1表达及组织学分型）的疗效及安全性。最短随访8.1个月时的中期分析显示，与单用化疗相比，联合组能够显著延长患者的OS（14.1个月*vs* 10.7个月，HR=0.69，*P*=0.000, 6）和PFS（6.8个月*vs* 5.0个月，HR=0.70；*P*=0.000, 1），并显著改善患者ORR（37.7% *vs* 25.1%）^[21]^。随访至少12.7个月时，联合治疗组与单用化疗组患者的中位OS分别为15.6个月和10.9个月（HR=0.66），1年的OS率分别为63%和47%；中位PFS则分别为6.7个月和5.0个月（HR=0.68）^[22]^。亚组分析显示，无论患者PD-L1表达高低、无论鳞癌或非鳞癌患者，联合治疗均显示出临床获益，有效改善患者总生存。在PD-L1阳性（≥1%）患者中，联合组与化疗组患者的中位OS分别为15.8个月和10.9个月（HR=0.64）；在PD-L1阴性（< 1%）患者中则分别为16.8个月和9.8个月（HR=0.62）^[22]^。联合治疗组与单用化疗组的3级-4级治疗相关不良事件的发生率分别为47%和38%^[22]^。

CheckMate 9LA最新公布的2年随访数据进一步肯定了双免疫联合化疗的持续生存获益^[23]^。无论患者PD-L1表达、组织学分型如何，双免疫联合化疗组患者2年时OS、PFS和DOR相关的获益均持续存在。双免疫联合化疗组与单用化疗组患者的中位OS分别为15.8个月和11.0个月（HR=0.72；其中PD-L1≥1%人群：15.8个月*vs* 10.9个月，HR=0.70；PD-L1 < 1%人群：17.7个月*vs* 9.8个月，HR=0.67），中位PFS分别为6.7个月和5.3个月（HR=0.67），中位DOR分别为13.0个月和5.6个月^[23]^。

既往临床研究^[14, 24]^表示，单纯免疫治疗对比化疗，生存曲线往往出现早期交叉的情况。而CheckMate 9LA研究中OS和PFS的生存曲线在早期即实现了分离，解决了生存曲线早期交叉的问题。双免疫联合2个周期的化疗能协同增加疗效，同时减轻联合标准化疗带来的不良反应。基于CheckMate 9LA研究数据，2020年美国食品药品监督管理局批准Nivolumab+Ipilimumab联合2个周期化疗用于晚期NSCLC患者一线治疗^[25]^。

此外，既往Nivolumab剂量多为3 mg/kg *Q2W*或240 mg *Q2W*，CheckMate 9LA采用360 mg *Q3W*，以便于临床操作及化疗方案的同步用药，从而降低临床错漏风险，减轻患者随访/就诊负担，增强患者依从性。双免疫联合方案用于一线治疗NSCLC的临床研究有效性数据总结见表 1。

**表 1:** 双免疫联合治疗一线用于NSCLC的有效性和安全性总结 Summary of the efficacy and safety of dual immunotherapy as first-line treatment in NSCLC

Study	Phase	Study design		Efficacy					Safety		Most common AEs
				ORR (%)	PFS (mon)	OS (mon)	DOR (mon)		Any AEs (%)	G3 or higher AEs	
CheckMate 012	I	N1 *Q2W*+I1 *Q6W*		33	5.6	NR	NR		73	40	The most frequently observed categories of treatment-related select adverse events were skin (39% in Ipilimumab *Q12W* cohort and 36% in Ipilimumab *Q6W* cohort), gastrointestinal (24% and 23%), and endocrine (11% and 21%)
		N3 *Q2W*+I1 *Q6W*		38	3.9	-	NR		74	31	
		N3 *Q2W*+I1 *Q12W*		47	8.1	-	NR		84	42	
CheckMate 568 (p1)	Ⅱ	N3 *Q2W*+I1 *Q6W*		30	4.2	-	NR		80	29	The most common treatment-related select AEs of any grade were skin reactions (30%), and the most common grade 3 to 4 treatment-related select AEs were gastrointestinal toxicities (5%)
CheckMate 568 (p2)	Ⅱ	Chemo*+ N 360 mg *Q3W*+ I1 *Q6W*		47	10.8	19.4	12.7		92	58	
CheckMate 227	Ⅲ	PD-L1≥1%	N3 *Q2W*+ I1 *Q6W*	36.4	5.1	17.1	23.2		77.2	35.5	The most common potentially immune-mediated AEs were cutaneous (34.0% any grade, 4.2% grade≥3), endocrine (23.8% any grade, 4.2% grade≥3), gastrointestinal (18.2% any grade, 2.4% grade≥3) and hepatic (15.8% any grade, 8.2% grade≥3)
			Chemo	30.0	5.6	14.9	6.7		83.7	36.4	
		PD-L1 < 1%	N3 *Q2W*+ I1 *Q6W*	27.3	5.1	17.2	18.0		75.7	27.0	
			Chemo	23.1	4.7	12.2	4.8		78.1	35.0	
CheckMate 817	ⅡIb/Ⅳ	PS 0-1	N240mg *Q2W*+ I1 *Q6W*	36	5.8	17	NR		77	35	Rates of any grade select TRAEs by category ranged from 1.3% (renal) to 28.4% (skin). The most common grade 3-4 select TRAEs by category were hepatic (4.6%), pulmonary (3.1%), and gastrointestinal (3.1%)
		PS=2 or PS=1 with≥1 selected comorbidities		24	3.9	9.9	13.8		67	28	
CheckMate 9LA	Ⅲ	Chemo+ N 360 mg *Q3W*+ I1 *Q6W*		38.0	6.7	15.8	13.0		92	48	The most common potentially immune-mediated AEs were cutaneous (40.5% any grade, 4.5% grade≥3), endocrine (25.7% any grade, 2.8 grade≥3), and gastrointestinal (23.3% any grade, 5.6% grade≥3) and hepatic (14.4% any grade, 4.4% grade≥3)
		Chemo		25.4	5.3	11.0	5.6		88	38	
*2 cycles of platinum-based doublet chemotherapy. I1: Ipilimumab 1 mg/kg; mon: month; N1: Nivolumab 1 mg/kg; N3: Nivolumab 3 mg/kg; AE: adverse event; Chemo: chemotherapy; DOR: duration of response; G: grade; NSCLC: non-small cell lung cancer; ORR: objective response rate; OS: overall survival; PD-L1: programmed cell death-ligand 1; PFS: progression free survival; PS: performance status; TRAEs: treatment related adverse events; NR: not reached.											

### 2.4 双免疫联合治疗的安全性

双免疫联合治疗尤其是Ipilimumab的安全性一直受到临床医师的重点关注。CheckMate 227和CheckMate 9LA等多项大型临床研究^[16-23]^均提示，双免疫联合治疗±化疗方案的任意不良反应与≥3级不良反应发生率均与对照化疗组类似。以CheckMate 227研究^[16]^为例，总体人群中联合治疗组和化疗组的3级-4级治疗相关不良反应发生率类似，分别为32.8%和36.0%；两组因治疗相关不良事件停药的患者比例分别为18.1%和9.3%，但长期生存随访结果提示这部分患者的生存获益并未受到影响，因治疗相关不良反应导致停药患者的4年OS率为44%。联合治疗组和化疗组分别有8例（1.4%）及6例（1.1%）患者发生治疗相关死亡。不同PD-L1水平患者的不良事件发生率与总体人群类似，且与既往研究一致。

双免疫联合治疗最常见的不良反应包括皮肤（任意级别和≥3级发生率分别为34%和4.2%）、内分泌（23.8%和4.2%）、胃肠道（18.2%和2.4%）和肝脏（15.8%和8.2%）相关不良事件^[16]^；双免疫联合化疗方案最常见的不良反应类型一致，分别为皮肤（任意级别和≥3级发生率分别为40.5%和4.5%）、内分泌（25.7%和2.8%）、胃肠道（23.3%和5.6%）和肝脏（14.4%和4.4%）^[22]^。总体而言，双免疫联合治疗单用及联合化疗的安全性均可控，且在长期随访过程中无新型安全性事件发生^[16-23]^。遵循早期检测、早期预防、及时处理、必要时暂停治疗的管理原则，多数不良反应均可得到有效缓解。因此，联合多学科综合管理、遵照现有的免疫相关不良事件管理共识或指南，及时检测并应对不良事件的发生，或可消除双免疫联合治疗的安全性顾虑。

## 3 生物标志物

迄今为止，免疫治疗的获益人群正不断增加，但尚未出现理想的生物标志物来精准预测疗效或最佳获益人群。无论是PD-L1阴性患者或低TMB/低微卫星不稳定性（microsatellite instability-low, MSI-L）患者，仍有部分可能从免疫治疗尤其是双免疫联合治疗中获益。因此，寻找理想的生物标志物或建立生物标志物综合评分网络迫在眉睫。而且双免疫治疗与免疫单药或者免疫联合化疗的生物标志物是否存在差异也值得探讨。生物标志物的联合应用及动态监测可能是未来探索的重要方向。

### 3.1 PD-L1

PD-L1仍是目前较为公认的免疫治疗预测性生物标志物。KEYNOTE-024研究的结果提示高PD-L1表达（PD-L1≥50%）的患者接受帕博利珠单抗治疗时的抗肿瘤效应显著更强；KEYNOTE-042研究也显示高PD-L1表达的患者接受帕博利珠单抗治疗时的生存获益优于化疗，而PD-L1表达在1%-49%之间的患者生存获益仅与化疗相当^[26]^。然而，CheckMate 017研究和OAK研究却提示，PD-L1并非在所有临床环境下对所有免疫治疗的疗效均是合适的预测性生物标志物，低PD-L1表达患者也有可能从Nivolumab或阿替利珠单抗治疗中同等获益（PD-L1阴性患者对免疫检查点抑制剂单药或联合治疗的ORR可达11%-20%）^[26]^。其中机制尚未明确，但推测可能有其他信号通路如表皮生长因子受体（epidermal growth factor receptor, EGFR）、MAPK或PI3K-AKT在肿瘤进展中发挥作用；反之，也可能是在治疗过程中有其他炎症反应性细胞因子[如γ干扰素（interferon γ, IFN-γ）等]诱导并刺激了PD-L1在肿瘤微环境中的动态表达^[26]^。CheckMate 227和CheckMate 9LA的结果进一步加深了PD-L1是否可以预测双免疫联合治疗疗效的顾虑，两项研究都表明无论PD-L1表达如何，双免疫或者双免疫联合化疗都比化疗组获得更显著的生存获益^[17, 22]^。

因此，PD-L1表达可能尚无法作为双免疫联合治疗疗效预测的综合性、独立性标志物^[2, 27]^。其有限性在于：①PD-L1检测方法和抗体尚未标准化或得到完美一致性；②PD-L1在肿瘤组织中的表达具有时间和空间上的异质性，研究^[27]^显示转移性NSCLC患者（*n*=398）接受免疫治疗时的PD-L1表达根据临床疗程的时间变化和解剖学部位均有所不同，如肾上腺、肝脏和淋巴结转移灶最高，骨及脑转移灶最低。选择不同活检部位时，PD-L1表达与免疫治疗的生存获益相关性也有所不同，如同一研究中，肺或远处转移灶标本的PD-L1表达与治疗反应和生存预后显著相关，但淋巴结转移灶标本的PD-L1表达与两者均不相关；③PD-L1阳性评分的标准和阈值也尚未能标准化；④其他免疫检查点包括CTLA-4、TIM-3、LAG-3等均可能产生PD-L1表达无法预测的肿瘤逃逸效应，或其抑制剂也会存在PD-L1表达无法预测的抗肿瘤效应^[27]^。

### 3.2 TMB

2019年的世界肺癌大会上，KEYNOTE-189^[28]^和KEYNOTE-021^[29]^研究显示TMB无法预测免疫治疗联合化疗的疗效；但CheckMate 227前期结果揭示了双免疫联合治疗在高TMB人群中获益不俗^[30]^。在经FoundationOne CDx试剂盒检测为高TMB（≥10个突变/Mb）的患者中，Nivolumab+Ipilimumab组和化疗组的中位PFS分别为7.2个月和5.5个月（HR=0.58, *P* < 0.001），ORR分别为45.3%和26.9%，1年无进展生存率分别为42.6%和13.2%^[30]^。尽管该研究中TMB无法有效预测患者OS，但可独立预测患者PFS，这一结果与既往研究中已印证的TMB对PFS的预测作用（如KEYNOTE-028研究，帕博利珠单抗）一致。患者报告的结局也显示，高TMB患者中，治疗12周时症状恶化的患者较化疗组显著较少（22.3% *vs* 35.0%），双免疫治疗组患者出现首次症状恶化的时间延迟^[30]^。因此，TMB或可作为双免疫联合治疗的生物标志物之一，但鉴于TMB作为生物标志物的探索尚在初期，尽管部分研究中TMB有较好的预后预测能力，目前各大指南尚未采纳TMB作为免疫治疗的用药决策协助指标或疗效预测指标。

### 3.3 其他潜在的生物标志物

恶性胸膜间皮瘤中的双免疫治疗相关研究（CheckMate 743）发现高4基因炎症标签评分（4-gene inflammatory signature score）与双免疫治疗组生存获益显著改善相关，高评分和低评分人群接受双免疫治疗的中位OS分别为21.8和16.8个月（HR=0.57）；但在化疗组并未观察到这种相关性（HR=1.14），提示基因炎症标签评分可能成为双免疫联合治疗潜在的预测性生物标志物^[31]^。

此外，临床前研究提示，双免疫联合治疗的生存获益与肿瘤内部的CD8^+^ T细胞或肿瘤浸润性淋巴细胞（tumor infiltrating lymphocyte, TIL）的浸润程度相关^[6]^。在三阴性乳腺癌、黑色素瘤和滑膜肉瘤中，CD8^+^ T细胞密度与TIL浸润程度高度相关，并可预测患者的病理学完全缓解率，因此，CD8^+^ T细胞密度/TIL或可成为未来的生物标志物探索方向之一。

## 4 新兴双免疫联合方案的探索

### 4.1 PD-1/PD-L1抑制剂+TIGIT抑制剂

研究显示新兴免疫检查点TIGIT参与CD8^+^ T细胞^[32]^和自然杀伤（natural kill, NK）细胞^[33]^的耗竭，因此，联合抑制TIGIT可能使更多的CD8^+^ T细胞被激活，并增强NK细胞的肿瘤杀伤能力，无论是在炎症型肿瘤（inflamed tumor，或称热肿瘤）或沙漠型肿瘤（immune desert，或称冷肿瘤）中，都可能增强PD-1/PD-L1抑制剂的疗效^[33]^。CITYSCAPE研究应用TIGIT抑制剂Tiragolumab联合PD-L1抑制剂阿替利珠单抗治疗PD-L1阳性NSCLC患者，针对PD-L1 TPS≥50%患者亚组的探索性分析显示^[34]^，Tiragolumab+阿替利珠单抗组与安慰剂+阿替利珠单抗组的ORR分别为66%和24%，PFS分别为未到达和4.11个月（HR=0.30），联合治疗获益明显。在小细胞肺癌患者中开展的CITYSCAPE-02研究（Tiragolumab+阿替利珠单抗）亦在进行之中。

### 4.2 PD-1/PD-L1抑制剂+白介素1β（interleukin-1β, IL-1β）抑制剂

临床前小鼠模型研究^[35]^提示IL-1β与免疫抑制微环境相关，IL-1β缺陷小鼠的抗肿瘤免疫力增强，包括激活CD8^+^淋巴细胞表达IFN-γ、肿瘤坏死因子α（tumor necrosis factor α, TNF-α）和颗粒酶B，并可观察到该类活性CD8^+^淋巴细胞浸润肿瘤从而诱导肿瘤退缩。临床前研究^[35]^证据显示IL-1β抑制剂与PD-L1抑制剂可起到协同作用，较单用两药时均更为显著抑制肿瘤进展（*P* < 0.05）。但近期，IL-1β抑制剂Canakinumab联合帕博利珠单抗加铂类双药化疗一线治疗局部晚期或转移性NSCLC的Ⅲ期临床研究CANOPY-1被宣告未达到改善OS和PFS的主要终点^[36]^。Canakinumab对比安慰剂术后辅助治疗Ⅱ期-Ⅲb期NSCLC的Ⅲ期研究CANOPY-A正在进行之中。

### 4.3 PD-1/PD-L1抑制剂+LAG-3抑制剂

LAG-3（lymphocyte-activation-gene-3 protein）是一种由*LAG-3*基因编码的免疫球蛋白，主要表达在活化的T细胞、NK细胞和浆细胞样树突状细胞的免疫检查点分子，其主要配体为组织相容性复合体Ⅱ（major histocompatibility complex Ⅱ, MHC-Ⅱ），以与CTLA-4和PD-1相似的方式负性调节T细胞的增殖和活化，并使CD8^+^ T细胞维持耐受状态^[37]^。临床前研究提示，单独或与PD-1抗体联合抑制LAG-3分子均可使T细胞重新恢复细胞毒性，从而抑制肿瘤生长^[37, 38]^。Relatlimab是一种抗LAG-3的单克隆抗体。一项评估Relatlimab联合Nivolumab对比Nivolumab单药一线治疗黑色素瘤的随机、双盲、Ⅱ期/Ⅲ期研究CA224-047证实抗LAG-3和PD-1联合能为晚期黑素瘤患者带来显著临床获益，意向治疗（intent to treat, ITT）人群双免疫联合组和Nivolumab单药组中位PFS分别为10.12个月和4.63个月（HR=0.75, *P*=0.005, 5）^[39]^。抗LAG-3和PD-1联合治疗NSCLC的相关研究目前正在进行。

### 4.4 PD-1/PD-L1抑制剂+SEMA4D抑制剂

SEMA4D与受体丛状蛋白B1和CD72结合，通过多种信号转导途径，在神经系统的轴突导向、免疫系统中T/B细胞的活化和免疫调节中发挥关键作用。在临床前肿瘤微环境中，炎性细胞和肿瘤细胞表达SEMA4D调节髓细胞和淋巴细胞的浸润、空间分布和活性，并可能形成屏障阻碍免疫细胞浸润肿瘤组织，从而形成免疫排除型（immune-excluded，或称非浸润型）肿瘤微环境^[40]^。在动物模型研究中，用抗体阻断SEMA4D可延缓肿瘤生长，并促进持久的肿瘤排斥反应^[41]^。

CLASSICAL-Lung研究旨在评估SEMA4D抑制剂Pepinemab与PD-L1抑制剂Avelumab治疗晚期NSCLC的安全性、耐受性和疗效（*n*=62）^[42]^。初步结果显示，对于既往未接受过免疫治疗的患者，Pepinemab+Avelumab联合治疗的疾病控制率（部分缓解+疾病稳定患者）达到81%，其中5/21例达到部分缓解，12/21例达到疾病稳定。对于PD-L1表达阴性和弱阳性患者联合方案也显示出疗效。此外，对于既往免疫治疗后出现疾病进展的患者，该联合方案的疾病控制率也达到59%，其中2/29例达到部分缓解，15/29例达到疾病稳定。对患者的动态追踪活检标本分析显示，SEMA4D抑制剂可改变免疫微环境的免疫平衡，增加T细胞渗透性和抗肿瘤活性，对联合方案有治疗反应的患者肿瘤样本中均可以发现T细胞浸润增加和肿瘤细胞的减少^[42]^。

## 5 总结

免疫治疗的出现已改变包括NSCLC在内的多种恶性肿瘤的治疗格局，尽管患者生存获益不断改善，但研究者仍未停止探索脚步。双免疫联合治疗进一步拓展了晚期NSCLC患者的治疗选择，基于其对免疫系统的双重调节/多通路阻滞作用，不仅可能使更多单药预期覆盖范围外（如PD-L1阴性）的患者获益，更可能通过长期调控并使肿瘤试图破坏的免疫系统维持正常化（normalization）^[43]^，从而使患者得到持久的治疗反应。同时，作为一种新兴治疗方案，双免疫联合治疗可能推动新兴生物标志物的探索，以寻找到更为精确的最佳获益人群。联合治疗的长期临床管理（包括剂量探索与不良反应管理等）亦需在临床实践中不断调整，并联合各学科协助形成标准化流程。此外，有关各种联合方案的开拓，包括新兴药物的加入和现有药物的新型联合，也将是未来的重要研究方向。
